# Identification of DDIT4 as a potential prognostic marker associated with chemotherapeutic and immunotherapeutic response in triple-negative breast cancer

**DOI:** 10.1186/s12957-023-03078-7

**Published:** 2023-06-30

**Authors:** Xuanzhao Chen, Zeyan Li, Meihua Liang, Ziyang Zhang, Di Zhu, Biyun Lin, Renyu Zhou, Yuanzhi Lu

**Affiliations:** 1 The Center of Pathological Diagnosis and Research, Affiliated Hospital of Guangdong Medical University, Zhanjiang, China; 2 Renmin Hospital of Wuhan University, Wuhan, Hubei China; 3 Guangzhou Huayin Medical Laboratory Center, Ltd., Guangzhou, China; 4 Department of Clinical Pathology, First Affiliated Hospital of Jinan University, Guangzhou, China; 5 School of Medicine, Jinan University, Guangzhou, China

**Keywords:** TNBC, Bioinformatics, Prognosis, Targeting therapy, Immune microenvironment

## Abstract

**Background:**

Triple-negative breast cancer (TNBC) is the most heterogenous and aggressive subtype of breast cancer. Chemotherapy remains the standard treatment option for patients with TNBC owing to the unavailability of acceptable targets and biomarkers in clinical practice. Novel biomarkers and targets for patient stratification and treatment of TNBC are urgently needed. It has been reported that the overexpression of DNA damage-inducible transcript 4 gene (DDIT4) is associated with resistance to neoadjuvant chemotherapy and poor prognosis in patients with TNBC. In this study, we aimed to identify novel biomarkers and therapeutic targets using RNA sequencing (RNA-seq) and data mining using data from public databases.

**Methods:**

RNA sequencing (RNA-Seq) was performed to detect the different gene expression patterns in the human TNBC cell line HS578T treated with docetaxel or doxorubicin. Sequencing data were further analyzed by the R package “edgeR” and “clusterProfiler” to identify the profile of differentially expressed genes (DEGs) and annotate gene functions. The prognostic and predictive value of DDIT4 expression in patients with TNBC was further validated by published online data resources, including TIMER, UALCAN, Kaplan–Meier plotter, and LinkedOmics, and GeneMANIA and GSCALite were used to investigate the functional networks and hub genes related to DDIT4, respectively.

**Results:**

Through the integrative analyses of RNA-Seq data and public datasets, we observed the overexpression of DDIT4 in TNBC tissues and found that patients with DDIT4 overexpression showed poor survival outcomes. Notably, immune infiltration analysis showed that the levels of DDIT4 expression correlated negatively with the abundance of tumor-infiltrating immune cells and immune biomarker expression, but correlated positively with immune checkpoint molecules. Furthermore, DDIT4 and its hub genes (ADM, ENO1, PLOD1, and CEBPB) involved in the activation of apoptosis, cell cycle, and EMT pathways. Eventually, we found ADM, ENO1, PLOD1, and CEBPB showed poor overall survival in BC patients.

**Conclusion:**

In this study, we found that DDIT4 expression is associated with the progression, therapeutic efficacy, and immune microenvironment of patients with TNBC, and DDIT4 would be as a potential prognostic biomarker and therapeutic target. These findings will help to identify potential molecular targets and improve therapeutic strategies against TNBC.

## Introduction

Triple-negative breast cancer (TNBC) is the most aggressive subtype of breast cancer. It is characterized by the absence of estrogen receptor (ER), progesterone receptor (PR), and human epidermal growth factor receptor 2 (HER-2 receptor 2, HER-2) expression and accounts for 10–20% of all breast cancers [1]. Because established therapeutic targets for TNBC remain unidentified, non-specific and toxic chemotherapy is the only standard treatment option for patients with TNBC [2, 3].

In the past decade, Lehmann et al. identified six molecular subtypes of TNBC (TNBC type-6) based on the mRNA expression profiles [4], namely the basal-like 1/2 (BL1 and BL2), immunomodulatory (IM), mesenchymal (M), mesenchymal stem-like (MSL), and luminal androgen receptor (LAR) types, and different TNBC types were found to demonstrate differential sensitivity to standard-of-care neoadjuvant or adjuvant chemotherapy with anthracycline and cyclophosphamide followed by taxane (ACT) [5]. More recently, evidence from a comprehensive analysis combining the genomic and transcriptomic landscape of TNBC also indicated that TNBCs may classified into four transcriptome-based subtypes, namely luminal androgen receptor (LAR), immunomodulatory (IM), basal-like immune-suppressed (BLIS), and mesenchymal-like (ML) [6, 7]. This TNBC type may be feasibly classified using immunohistochemical surrogate biomarkers with AR, CD8, FOXC1, and DCLK1 [8]. Accordingly, an umbrella clinical trial was conducted for patients with refractory TNBC who received standard chemotherapy, including anthracyclines, taxanes, platinum, vinorelbine, capecitabine, and gemcitabine. The preliminary results of this study showed that the highest objective response rate (ORR) was achieved in intention-to-treat (ITT) TNBC patients treated with anti PD-1 and nab-paclitaxel or anti-VEGFR [9]. The results from the KEYNOT-355 clinical trial demonstrated that patients with advanced TNBC, with the tumor expression of PD-L1 and a combined positive score (CPS) of 10 or more significantly benefited from the chemotherapy plus pembrolizumab with longer overall survival (OS) than that achieved with chemotherapy alone [10]. Moreover, a complete pathological response was significantly higher among patients with early TNBC treated with pembrolizumab plus neoadjuvant chemotherapy than among patients who underwent neoadjuvant chemotherapy with a placebo (KEYNOTE-522, NCT03036488) [11]. These data shed promising light on the clinical management of TNBC. However, primary results from the IMpassion131 (NCT03125902) clinical trial indicated that the combination of atezolizumab, an anti-PD-L1 antibody, with paclitaxel did not improve the PFS or OS vs*.* paclitaxel alone among patients with TNBC, despite no concern of safety and tolerability with longer follow-up [12]. The paradoxical results from clinical trials reveal the limitations of the current system of transcriptome-based classification based on pool-tissue mRNA profiling to guide TNBC treatment.

Indeed, driver alterations have been recognized to be more streamlined and heterogenous, and over 80 to 90% of *TP53* pathogenic mutations were detected in primary and metastatic TNBC, respectively. Remarkably, the amplification of *MYC*, *PIK3CA*, *KRAS*, *BRAF*, *EGFR*, *CCNE1*, and *MDM2* and mutations of *CDKN2A/B* and *PTEN* are frequently detected in TNBC, which indicates that the co-activation of intrinsically oncogenic signaling networks predominantly drives the evolution of this disease [7, 13]. In addition, chemotherapy and/or radiotherapy may change the functions of tumor and stromal cells, such as by promoting PD-L1 expression, which results in immune evasion and resistance to anti-PD-1 immunotherapy [14, 15]. In accordance with this finding, several recent studies also sought to classify TNBC based on immunogenomic profiling and/or metabolic-pathway subtyping and attempt to provide identifiable biomarkers for guiding treatment [16, 17].

However, the usage of the current classification system for TNBC is less useful in routine clinical practice owing to the complex technology requirements of the platform and low affordability. Therefore, novel biomarkers need to be identified to stratify patients with TNBC for targeting therapy, immunotherapy, or combined chemotherapy. Reportedly, the abrogation of metabolic activity triggered by the aberrant activation of the PI3K/AKT/mTOR pathway in cancer cells may result in the deregulation of genes involved in DNA damage and immune response, including DNA damage inducible transcript 4 (DDIT4). The *DDIT4* gene located in chromosome 10 (10q22.1) is 2.1 kb in length, containing three exons and two introns [18]. DDIT4 protein localized mainly in the cytoplasm regulates the mTOR activity by tuberous sclerosis complex (TSC1/TSC2 complex) [19, 20]. Confocal microscopy confirms that DDIT4 and mitochondria had obvious co-localization, and DDIT4 is ubiquitously expressed at very low levels under normal physiological conditions [18, 21]. Mechanistically, the expression of DDIT4 is induced by the activation of multiple cellular stress pathways, such as hypoxia, energy depletion, endoplasmic reticulum stress response, and DNA damage by etoposide and arsenite [22]. A correlation between DDIT4 expression and poor survival was found in specific tumor types including ovarian cancer [23], breast cancer [24], lung cancer [25], and bladder urothelial carcinoma [26], which suggests that DDIT4 may be a cancer related protein and potential biomarker. Interestingly, the upregulation of DDIT4 in TNBC is associated with resistance to neoadjuvant chemotherapy and poor prognosis [27]. In this study, we perform RNA-seq of TNBC cells treated with docetaxel and doxorubicin and conduct a comprehensive analysis of RNA-seq data using bioinformatics tools. We demonstrated that DDIT4 may serve as a potential biomarker and therapeutic targets for TNBC.

## Materials and methods

### Establishment of docetaxel- and doxorubicin-treated cells

Docetaxel and doxorubicin were purchased from Sigma (Selleck, Shanghai, China). Human TNBC HS578T cells were purchased from the American Type Culture Collection (ATCC, Shanghai, China). Cells were cultured in RPMI-DMEM (Gibco, USA) with 10% fetal bovine serum (FBS), 1% penicillin, and 1% streptomycin at 37 °C with 5% CO_2_. The HS578T cells were seeded in 6-well culture plates at 2.5 × 10^5^ cells/well and were treated with 2 μM docetaxel or doxorubicin for 24 h.

### RNA-Seq

RNA was isolated and purified using TRIzol (Life, cat.265709, CA, USA) in accordance with the manufacturer’s protocol. RNA purity was assessed using the NanoPhotometer® spectrophotometer (IMPLEN, CA, USA). One microgram of RNA per sample was used as the input for RNA sample preparation. Sequencing libraries were generated using the NEBNext® UltraTM RNA Library Prep Kit for Illumina® (NEB, USA) in accordance with the manufacturer’s instructions, and index codes were added to attribute sequences to each sample. Briefly, mRNA was purified from total RNA using poly-T oligo-attached magnetic beads. Fragmentation was conducted using divalent cations at an elevated temperature in NEBNext first-strand synthesis reaction buffer (5X). The first-strand cDNA was synthesized using a random hexamer primer and M-MuLV reverse transcriptase (RNase H-). The second-strand cDNA synthesis was subsequently performed using DNA polymerase I and RNase H. The remaining overhangs were converted into blunt ends using exonuclease/polymerase. After the adenylation of the 3′ ends of DNA fragments, NEBNext Adaptor with a hairpin loop structure was ligated to prepare the samples for hybridization. To select cDNA fragments, preferentially spanning 250 ~ 300 bp, the library fragments were purified using the AMPure XP system (Beckman Coulter, Beverly, MA, USA). Following this, 3 µL of USER Enzyme (NEB, USA) was added with size-selected, adaptor-ligated cDNA at 37 °C for 15 min, followed by treatment for 5 min at 95 °C before PCR. PCR was performed using Phusion high-fidelity DNA polymerase, universal PCR primers, and Index (X) primer. The PCR products were purified (AMPure XP system), and the library quality was assessed on the Agilent Bioanalyzer 2100 system.

Finally, the clustering of the index-coded samples was performed on a cBot Cluster Generation System using TruSeq PE Cluster Kit v3-cBot-HS (Illumina) according to the manufacturer’s instructions. Following this, the library preparations were sequenced on an Illumina NovaSeq platform by Shanghai Genechem Co., Ltd. (Shanghai, China).

### Data quality control

Raw data (raw reads) in the fastq format were first processed using in-house perl scripts. In this step, clean data (clean reads) were obtained by removing reads containing adapter or ploy-N. Concurrently, the Q20, Q30, and GC contents were calculated from the clean data. The downstream analyses were based on clean data with high quality.

### GEO database

The GEO database is a high-throughput microarray and sequence functional genomic database (https://www.ncbi.nlm.nih). In this study, the GSE62931 dataset included data from 53 TNBC and 53 non-TNBC (ER + /PR +) samples.

### Differential gene expression analysis

Prior to the differential gene expression analysis, for each sequenced library, the read counts were adjusted using the edgeR program package through one scaling normalized factor. Differential expression analysis in two conditions was performed using the edgeR R package (3.18.1). The *P* values were adjusted using the Benjamini–Hochberg method. A corrected *P* value of 0.05 and absolute foldchange of 2 were set as the threshold for significantly differential expression.

### Functional annotation and pathway enrichment analysis

Gene Ontology (GO) enrichment analysis of differentially expressed genes was implemented using the clusterProfiler R package [28], in which the gene length bias was corrected. GO terms with a corrected *P* value less than 0.05 were considered significantly enriched by the differentially expressed genes. KEGG is a database resource for understanding the high-level functions and utilities of the biological system, such as the cell, organism, and ecosystem, from molecule-level information, especially large-scale molecular datasets generated via genome sequencing and other high-through put experimental technologies (http://www.genome.jp/kegg/). We used the clusterProfiler R package to test the statistical enrichment of differentially expressed genes in KEGG pathways.

### UALCAN

The UALCAN platform is an online portal based on The Cancer Genome Atlas (TCGA) that allows users to conduct comprehensive analysis of gene expression [29]. We determined the expression level of DDIT4 in breast cancer based on different clinicopathological characteristics using UALCAN data. *P* < 0.05 was regarded as an indicator of statistically significant results.

### Kaplan–Meier plotter

The Kaplan–Meier plotter (https://www.kmplot.com) is a database that can be used to investigate the associations between key genes and prognosis for breast cancer, ovarian cancer, lung cancer, and gastric cancer [30]. According to the media of DDIT4 expression, patients with breast cancer were divided into a low-expression group and a high-expression group, and the overall survival (OS), post-progression survival (PPS), distant metastasis-free survival (DMFS), and recurrence-free survival (RFS) rates were further analyzed.

### TIMER analysis

The TIMER database was used to estimate the number of tumor-infiltrating immune cells (TIICs) in different cancer types using samples from the TCGA (https://cistrome.shinyapps.io/timer/) database [31]. We used this database to assess expression levels of DDIT4 in different tumor types and explored the relationship between the expression of DDIT4 and the abundance of immune infiltrates in TNBC and breast cancer considering *p* < 0.05 as the cutoff for statistical significance.

### Relationship between the expression of immune checkpoint-related genes and that of DDIT4

Breast cancer RNA-seq-based gene expression data (“Level_3_HTSeq-FPKM _normalized”) were obtained from TCGA (https://portal.gdc.cancer.gov/). FPKM data were further converted into TPM data for correlation analysis between the expression of DDIT4 and immune checkpoint-related genes of interest. Analyses were conducted using R v3.6.3 and the software packages ggplot2 and heatmap. In all, the expression patterns of ten immune checkpoint-related genes were evaluated using Spearman correlation, and *p* < 0.05 was considered significant.

### LinkedOmics

LinkedOmics (http://www.linkedomics.org) hosts multi-omics data and clinical data from 32 different cancer types and 11,158 individuals obtained from the TCGA project [32]. We identified the genes whose expression was significantly correlated with DDIT4 expression in the LinkedOmics dataset and constructed a heatmap of the top 50 correlated genes. Pearson’s correlation test was used for statistical analysis, and *P* < 0.05 was considered statistically significant.

### GeneMANIA

GeneMANIA (https://www.genemania.org) was used to predict protein–protein interaction (PPI) networks and the potential function of DDIT4 [33]. After the hub genes were identified using LinkedOmics, we determined their potential functions using GeneMANIA. We also used GeneMANIA to determine the association between the expression of DDIT4 and hub genes.

### GSCALite

GSCALite is a bioinformatics platform for gene set cancer analysis, offering various types of analyses, including methylation, cancer-related pathway, and miRNA network analyses [34]. GSCALite was used for pathway activity analysis in this study using the TCGA sample.

### Statistical analysis

The gene expression level thresholds of |log2 fold change|> 1.0 and false discovery rate (FDR < 0.05) were used. *p* value < 0.05 was considered to indicate significant difference, and a survival analysis *p* value < 0.05 was considered to indicate significant influence prognosis.

## Results

### Identification and functional characterization of upregulated DEGs in docetaxel- and doxorubicin-treated TNBC cells

**Fig. 1:**
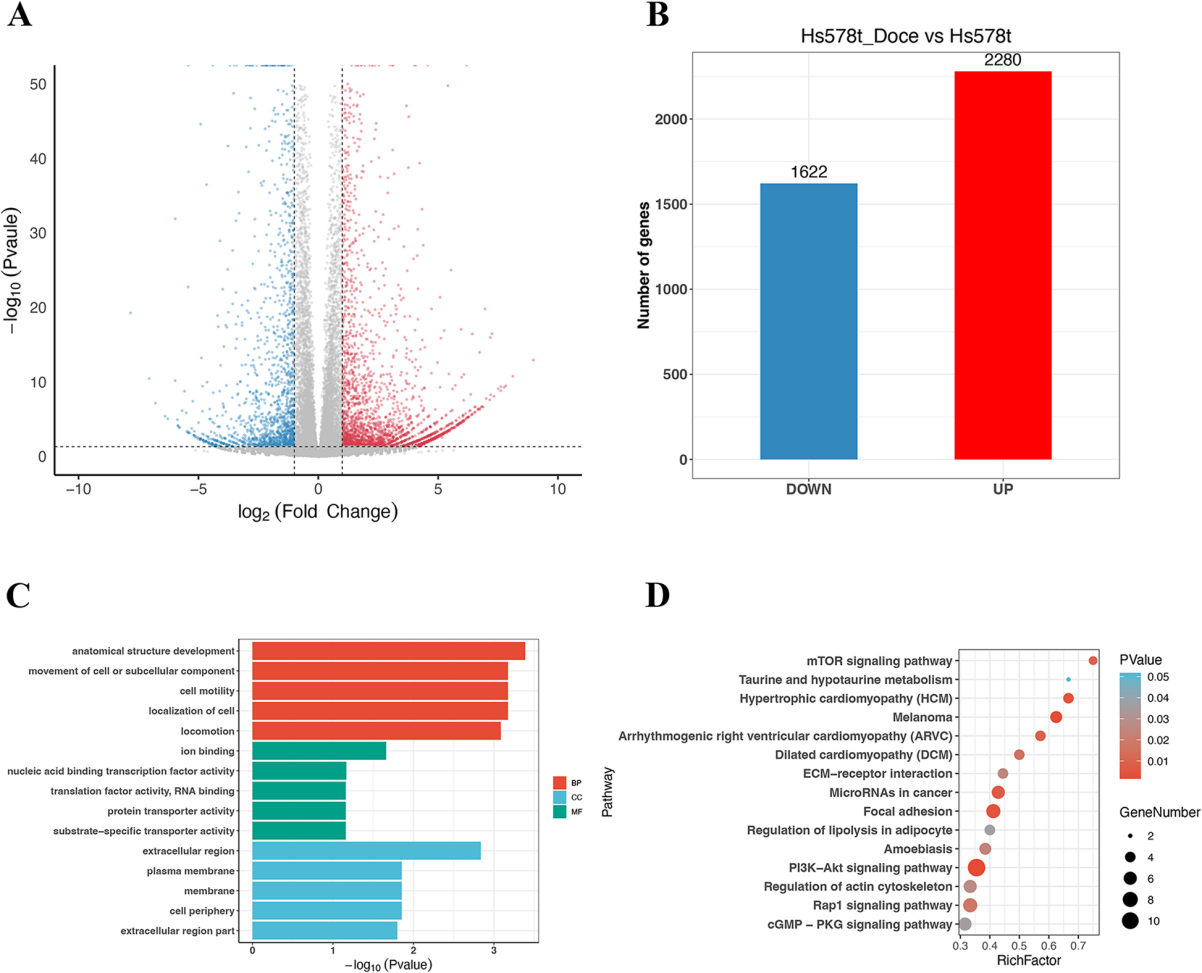
Identification and characterization of DEGs from the HS578T_Doce vs HS578T data. **A** Volcano plot of DEGs between docetaxel-treated cells and parental cells. The red dots represent significantly upregulated DEGs; the blue dots represent DEGs that were downregulated; the black dots indicate no significant difference (*P* < 0.05 and |log2FC|> 1.0 as the threshold). **B** Distribution of DEGs of significance in docetaxel-treated cells. The top five GO terms (**C**) and KEGG enriched pathways (**D**) of significantly DEGs are indicated. BP, biological process; CC, cell component; MF, molecular function

To identify the potential genes related to sensitivity to docetaxel and doxorubicin in TNBC, we first performed differential expression analysis using the RNA-seq data of HS578T cells treated with docetaxel or doxorubicin. The results showed the presence of 3902 DEGs in docetaxel-treated HS578T cells (Fig. 1A), of which 2280 DEGs were found to be significantly upregulated and 1622 DEGs were downregulated (Fig. 1B).

To explore the underlying biological function and signaling pathways, functional enrichment analysis for these DEGs was performed as previously described. Specifically, the BP group genes were enriched in anatomical structure development, movement of cell or subcellular component, cell motility, and localization of cell. In addition, the CC group genes were primarily related to extracellular region, plasma membrane, membrane, and cell periphery. The MF group genes were primarily enriched in ion binding, nucleic acid binding transcription factor activity, translation factor activity, RNA binding, and protein transporter activity (Fig. 1C). The KEGG pathway analysis of the DEGs showed that the mTOR signaling pathway, microRNAs in cancer, and PI3K/AKT signaling pathway were most significantly enriched (Fig. 1D).

**Fig. 2:**
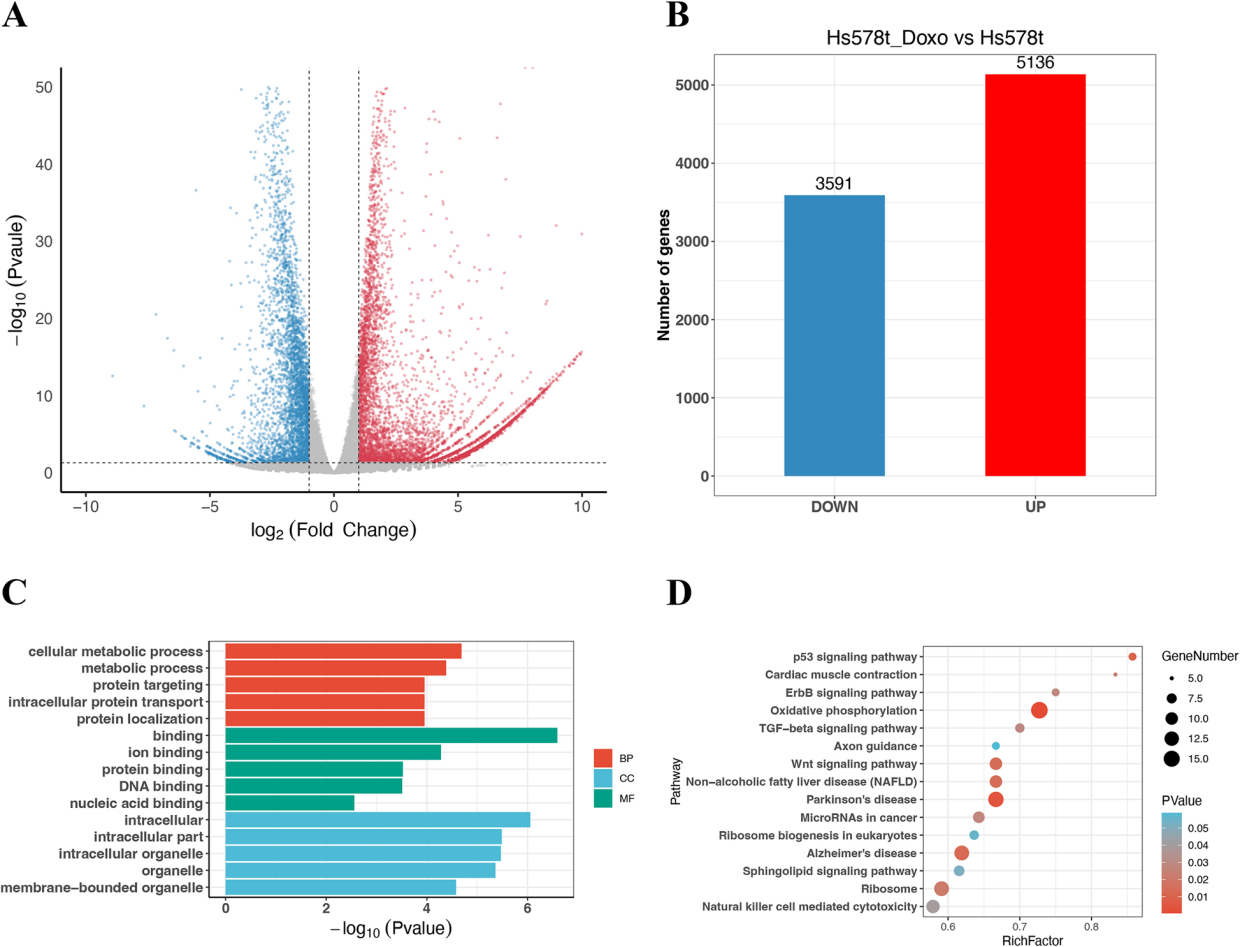
Identification and characterization of DEGs from the HS578T_Doxo vs HS578T data. **A** Volcano plot of DEGs between doxorubicin-treated cells and parental cells. The red dots represent significantly upregulated DEGs; the blue dots represent DEGs that were downregulated; the black dots indicate no significant difference (*P* < 0.05 and |log2FC|> 1.0 as the threshold). **B** Distribution of DEGs of significance in doxorubicin-treated cells. The top five GO terms (**C**) and KEGG enriched pathways (**D**) of significantly DEGs are indicated. BP, biological process; CC, cell component; MF, molecular function

Concurrently, 8727 DEGs were identified in doxorubicin-treated HS578T cells (Fig. 2A), of which 5136 DEGs were found to be significantly upregulated and 3591 DEGs were downregulated (Fig. 2B). The findings indicated that the expression of these DEGs was strongly associated with cellular metabolic process, protein binding, DNA binding, and membrane − bounded organelle (Fig. 2C). As shown in Fig. 2D, the DEGs were enriched in oxidative phosphorylation, p53 signaling pathway, and Wnt signaling pathway.

### Validation of DDIT4 expression correlates with chemotherapy in TNBC

**Fig. 3:**
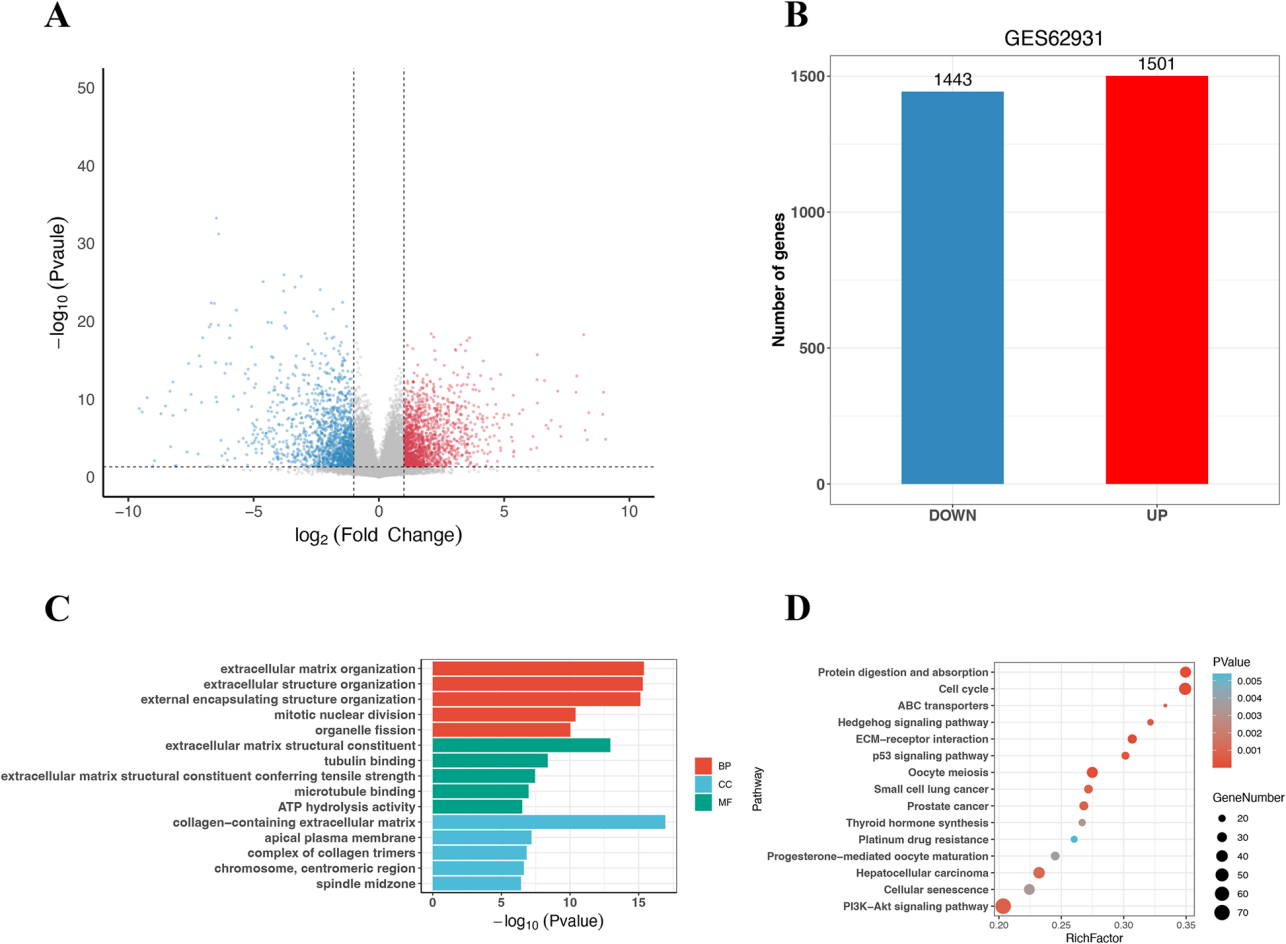
Identification and characterization of DEGs from the GSE62931 dataset. **A** Volcano plot of DEGs between TNBC samples and non-TNBC (ER + /PR +) samples. The red dots represent significantly upregulated DEGs; the blue dots represent DEGs that were downregulated; the black dots indicate no significant difference (*P* < 0.05 and |log2FC|> 1.0 as the threshold). **B** Distribution of DEGs of significance in TNBC tissues. The top five GO terms (**C**) and KEGG enriched pathways (**D**) of significantly DEGs are indicated. BP, biological process; CC, cell component; MF, molecular function

**Fig. 4:**
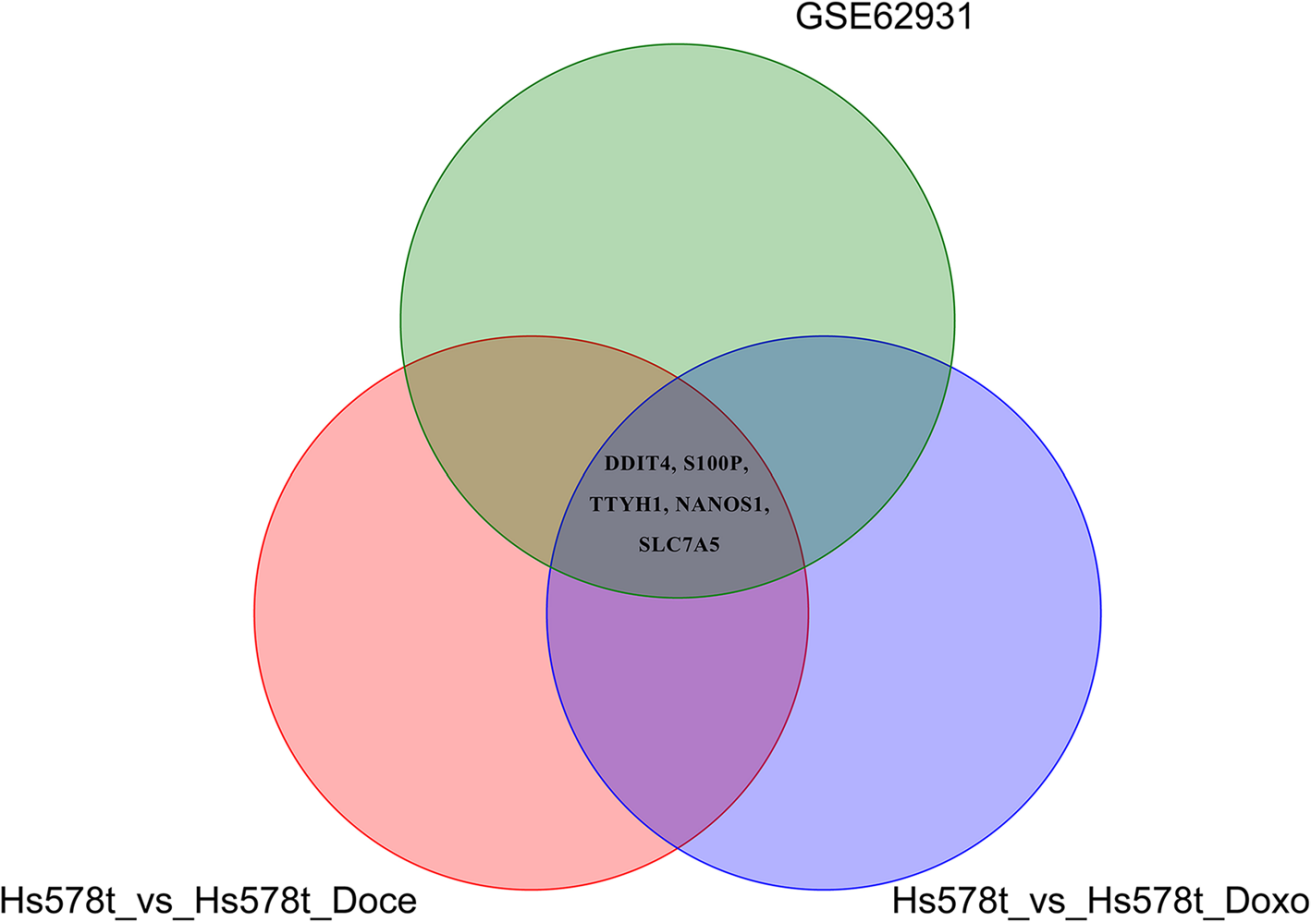
Venn diagram of DEGs from theHS578T_Doce vs HS578T, HS578T_Doxo vs HS578T, and GSE62931 datasets

To investigate more sensitive targets and verify the reliability of the results, we retrieved the GSE62931 datasets, which included TNBC and ER + /PR + samples. As shown in Fig. 3A, 2944 DEGs were identified, among which 1501 DEGs were upregulated and 1443 DEGs were significantly downregulated in TNBC cells (Fig. 3B). The GO terms showed that the DEGs were primarily related to collagen-containing extracellular matrix (Fig. 3C) and were enriched in cell cycle pathway, EMC-receptor interaction, and P53 and PI3K/AKT signaling pathway (Fig. 3D). Upon comparing the DEGs significantly upregulated in the three abovementioned gene sets (Fig. 4), five genes were identified, namely DDIT4, S100P [35], TTYH1 [36], NANOS1 [37], and SLC7A5 [38]. The expression of these genes was previously reported to be associated with the development of breast cancer. Limited data are available on DDIT4 expression in the context of chemotherapy and immunotherapy resistance in TNBC. Therefore, we further chose DDIT4 as the potential target gene of interest in this study.

### DDIT4 as a key indicator of the chemotherapeutic response in TNBC

**Fig. 5:**
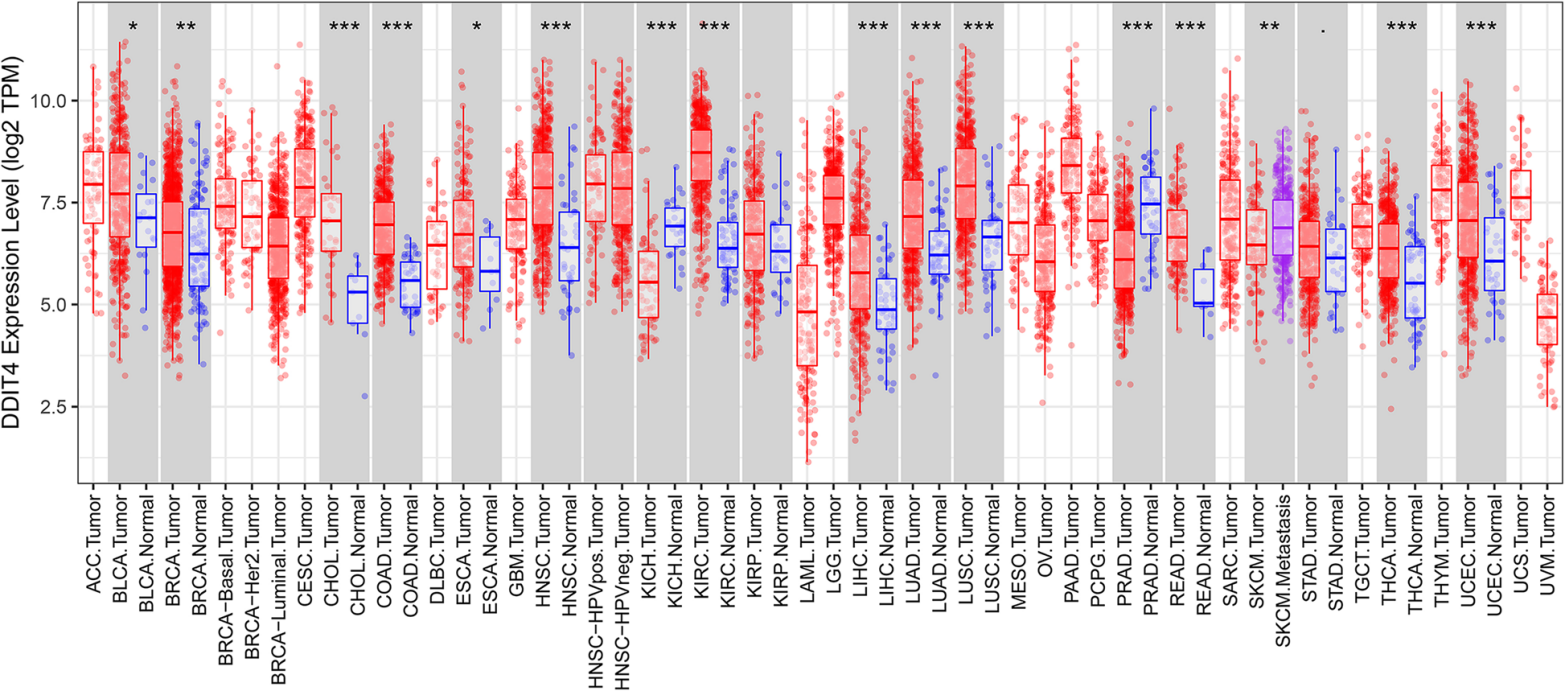
The expression level of DDIT4 in different cancers compared with that in normal tissues in the TIMER database

**Fig. 6:**
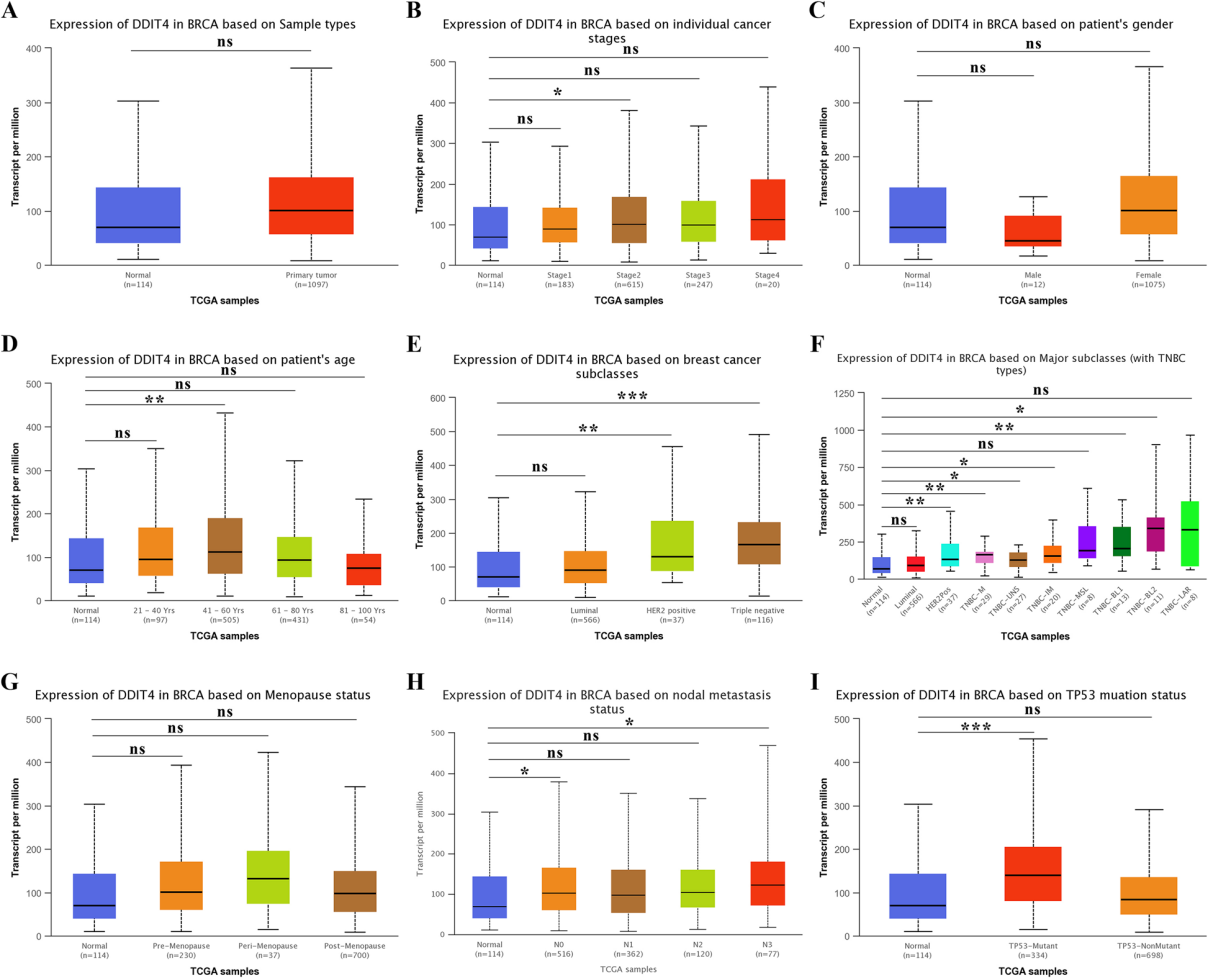
The expression levels of DDIT4 in breast cancer based on different clinical characteristics. *p < 0.05, **p < 0.01, ***p < 0.001

**Fig. 7:**
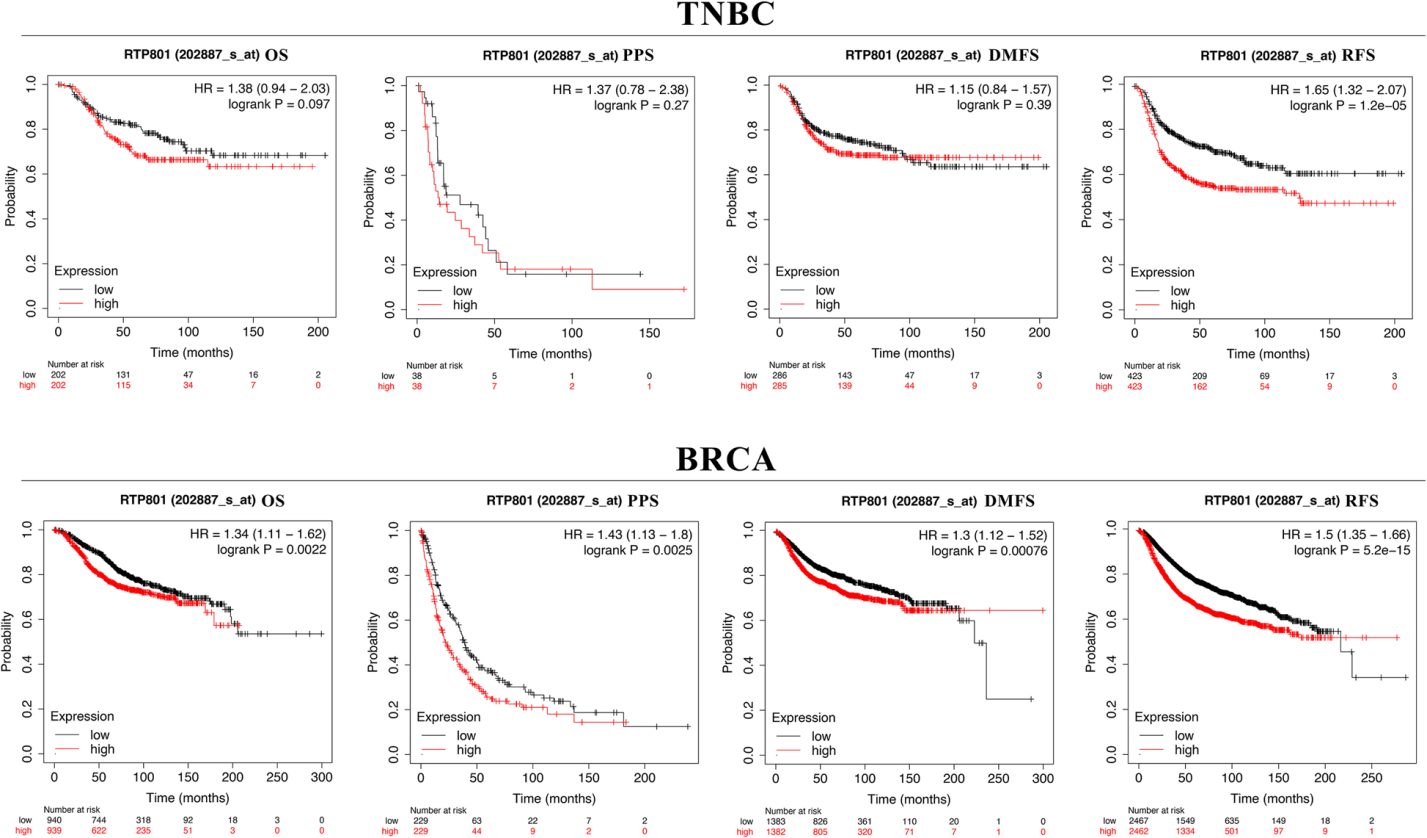
Survival analysis, indicated by the OS, PPS, DMFS, and RFS, based on DDIT4 expression in patients with TNBC and BC obtained from KM plotter

To determine the role of DDIT4 in TNBC, we first evaluated its expression levels and diagnostic and prognostic value in patients with TNBC. TIMER data revealed that the mRNA expression of DDIT4 was significantly higher in breast cancer tissues than in normal tissues (Fig. 5). Following this, we analyzed the transcription levels of DDIT4 based on the stages of breast cancer, patient gender, age, primary subtypes, major subclasses with TNBC, menopausal status, nodal metastasis status, and TP53 mutation status. The DDIT4 transcription levels in breast cancer samples were significantly higher than those in normal samples. In particular, TCGA data indicated a higher expression of DDIT4 in TNBC than in other subtypes of breast cancer (Fig. 6). Furthermore, we investigated the correlation between DDIT4 overexpression at the mRNA level and patient prognosis by plotting and comparing the OS, PPS, DMFS, and RFS of patients with BC and TNBC using the Kaplan–Meier plotter (Fig. 7). TNBC patients with high levels of DDIT4 expression had a shorter RFS (HR = 1.65 (1.32–2.07), *p* < 0.001). Further, DDIT4 overexpression was associated with worse OS (HR = 1.34 (1.11–1.62), *p* < 0.01), PPS (HR = 1.43 (1.13–1.8), *p* < 0.01), DMFS (HR = 1.3 (1.12–1.52), *p* < 0.001), and RFS (HR = 1.5 (1.35–1.66), *p* < 0.001) in breast cancer. Overall, the findings imply that the mRNA expression of DDIT4 was significantly correlated with the poor prognosis of patients with breast cancer and TNBC.

### Relationship between the transcriptional level of DDIT4 and immune cell infiltration in TNBC

**Fig. 8:**
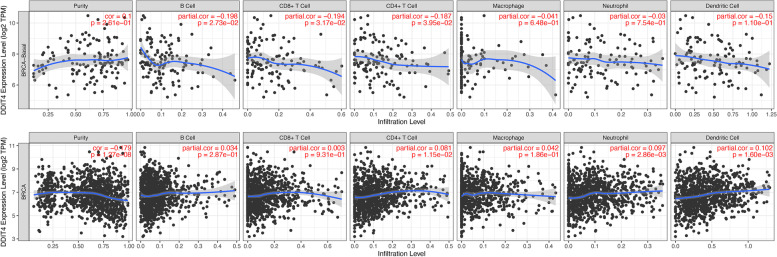
Relationship between the transcription DDIT4 and immune infiltrates in TNBC and BC

Immunotherapy has evolved into one of the most promising therapeutic regimens for TNBC [39]. However, the role of DDIT4 in immune infiltration in TNBC is unknown. Using the TIMER database, we further investigated the relationship between the transcriptional level of DDIT4 and immune infiltration. It was found that DDIT4 expression correlated negatively with the infiltration of B cells (Cor =  − 0.198, *p* < 0.05), CD8^+^ T cells (Cor =  − 0.194, *p* < 0.05), and CD4^+^ T cells (Cor =  − 0.187, *p* < 0.05). No significant association was observed between tumor purity and the infiltration of macrophages, neutrophils, and dendritic cells. We also analyzed the correlation between the DDIT4 transcription level and immune cell infiltration in breast cancer. The level of DDIT4 expression correlated positively with the infiltration of CD4^+^ T cells (Cor = 0.081, *p* < 0.05), neutrophils (Cor = 0.097, *p* < 0.01), and dendritic cells (Cor = 0.102, *p* < 0.01) and negatively with tumor purity (Cor =  − 0.179, *p* < 0.001) (Fig. 8).

**Fig. 9:**
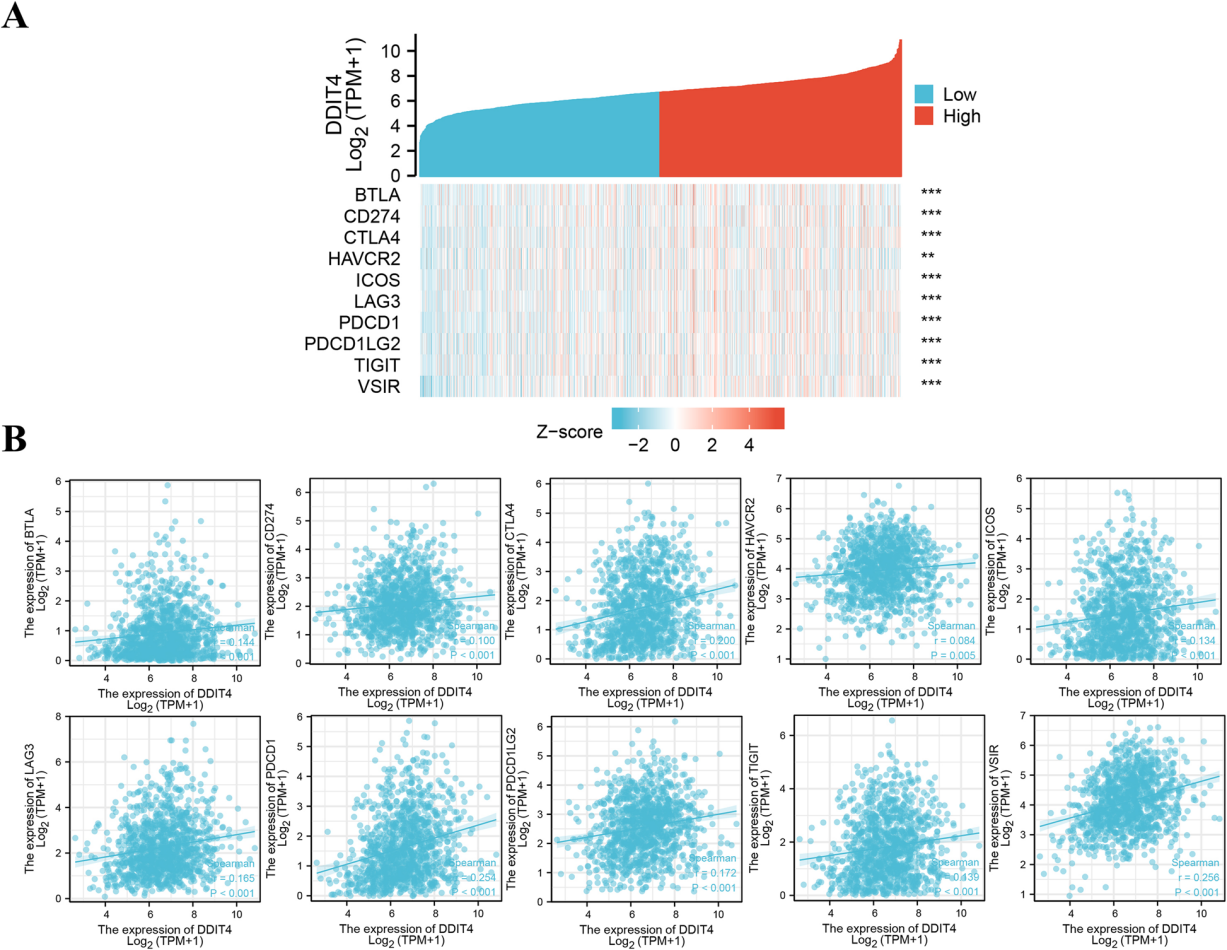
Relationship between the expression of ten immune checkpoint-related genes and DDIT4. Heatmap (**A**) and scatter plots (**B**) showing the relationship between the expression of DDIT4 and BTLA, CD274, CTLA4, HAVCR2, ICOS, LAG3, PDCD1, PDCD1LG2, TIGIT, and VSIR

The relationship between DDIT4 expression and immune marker expression was also analyzed. As shown in Table 1, the expression of DDIT4 correlated significantly with the expression of different genes with respect to the different immune subset cells in TNBC. The immune biomarkers identified were as follows: T cell markers, CD8A; B cells markers, CD19 and CD79A; neutrophil marker, CCR7; dendritic cell markers, HLA-DPB1, HLA-DPA1, and BDCA-1 (CD1C); Th1 markers, TNF-a (TNF); Treg markers, FOXP3 and CCR8. DDIT4 expression was negatively correlated with various immune cells. Further analysis of the relationship between the expression of ten immune checkpoint-related genes and DDIT4 showed that DDIT4 expression was positively correlated with BTLA, CD274, CTLA4, HAVCR2, ICOS, LAG3, PDCD1, PDCD1LG2, TIGIT, and VSIR expression in breast cancer (Fig. 9).

**Table 1:** Correlation analysis between DDIT4 and gene biomarkers of immune cells in TNBC (TIMER)

**Immune cell**	**Biomarker**		**None**		**Purity**
		**Cor**	***P* value**	**Cor**	***P* value**
CD8 + T cells	CD8A	− 0.179	3.40e − 02	− 0.177	4.53e − 02
	CD8B	− 0.158	6.25e − 02	− 0.155	7.99e − 02
T cells (general)	CD3D	− 0.151	7.58e − 02	− 0.145	1.02e − 01
	CD3E	− 0.16	5.91e − 02	− 0.156	7.80e − 02
B cells	CD2	− 0.167	4.81e − 02	− 0.172	5.25e − 02
	CD19	− 0.241	4.10e − 03	− 0.256	3.60e − 03
	CD79A	− 0.227	6.98e − 03	− 0.239	6.67e − 03
Monocytes	CD86	− 0.139	1.02e − 01	− 0.12	1.78e − 01
	CD115 (CSF1R)	− 0.125	1.43e − 01	− 0.094	2.91e − 01
TAMs	CCL2	− 0.046	5.87e − 01	− 0.026	7.72e − 01
	CD68	− 0.094	2.70e − 01	− 0.046	6.05e − 01
	IL10	− 0.114	1.80e − 01	− 0.078	3.80e − 01
M1 macrophages	INOS (NOS2)	− 0.037	6.64e − 01	− 0.079	3.72e − 01
	IRF5	− 0.082	3.34e − 01	− 0.075	4.00e − 01
	COX2 (PTGS2)	0.058	4.95e − 01	0.089	3.16e − 01
M2 macrophages	CD163	− 0.049	5.61e − 01	0.002	9.82e − 01
	VSIG4	− 0.03	7.23e − 01	0.024	7.91e − 01
	MS4A4A	− 0.142	9.41e − 02	− 0.11	2.18e − 01
Neutrophils	CD66b (CEACAM8)	− 0.048	5.74e − 01	− 0.095	2.88e − 01
	CD11b (ITGAM)	− 0.058	4.93e − 01	− 0.023	7.95e − 01
	CCR7	− 0.215	1.09e − 02	− 0.236	7.43e − 03
Natural killer cells	KIR2DL1	− 0.052	5.45e − 01	− 0.017	8.48e − 01
	KIR2DL3	0.027	7.51e − 01	0.095	2.87e − 01
	KIR2DL4	0.063	4.60e − 01	0.127	1.53e − 01
	KIR3DL1	− 0.076	3.72e − 01	− 0.055	5.34e − 01
	KIR3DL2	− 0.087	3.06e − 01	− 0.021	8.13e − 01
	KIR3DL3	− 0.072	4.01e − 01	− 0.055	5.37e − 01
	KIR2DS4	− 0.126	1.38e − 01	− 0.096	2.81e − 01
Dendritic cells	HLA-DPB1	− 0.211	1.26e − 02	− 0.206	1.94e − 02
	HLA-DQB1	− 0.16	5.84e − 02	− 0.144	1.04e − 01
	HLA-DRA	− 0.174	3.95e − 02	− 0.152	8.66e − 02
	HLA-DPA1	− 0.207	1.43e − 02	− 0.191	3.10e − 02
	BDCA-1 (CD1C)	− 0.233	5.52e − 03	− 0.216	1.42e − 02
	BDCA-4 (NPR1)	− 0.08	3.49e − 01	− 0.071	4.24e − 01
	CD11C (ITGAX)	− 0.162	5.55e − 02	− 0.151	8.92e − 02
Th1	T-bet (TBX21)	− 0.147	8.23e − 02	− 0.135	1.29e − 01
	STAT4	− 0.15	7.61e − 02	− 0.139	1.18e − 01
	STAT1	− 0.063	4.61e − 01	− 0.058	5.13e − 01
	IFN-g (IFNG)	− 0.094	2.70e − 01	− 0.07	4.30e − 01
	TNF-a (TNF)	0.168	4.75e − 02	0.2	2.39e − 02
Th2	GATA3	0.109	2.00e − 01	0.123	1.65e − 01
	STAT6	− 0.149	7.93e − 02	− 0.168	5.86e − 02
	STAT5A	0.024	7.81e − 01	0.035	6.93e − 01
	IL13	0	9.99e − 01	0.043	6.33e − 01
Tfh	BCL6	− 0.077	3.64e − 01	− 0.041	6.49e − 01
Th17	STAT3	0.135	1.11e − 01	0.154	8.26e − 02
	IL17A	0.012	8.91e − 01	0.029	7.45e − 01
Tregs	FOXP3	− 0.224	7.91e − 03	− 0.245	5.28e − 03
	CCR8	− 0.197	1.99e − 02	− 0.207	1.89e − 02
	STAT5B	− 0.08	3.48e − 01	− 0.084	3.48e − 01
	TGFb (TGFB1)	− 0.174	3.95e − 02	− 0.173	5.08e − 02
T cell exhaustion	PD-1 (PDCD1)	− 0.032	7.07e − 01	0.014	8.78e − 01
	CTLA4	− 0.118	1.64e − 01	− 0.092	3.01e − 01
	LAG3	− 0.031	7.13e − 01	− 0.004	9.68e − 01
	TIM-3(HAVCR2)	− 0.141	9.65e − 02	− 0.119	1.83e − 01
	GZMB	0.03	7.24e − 01	0.083	3.53e − 01

### Analysis of genes exhibiting co‑expression with DDIT4 in breast cancer

**Fig. 10:**
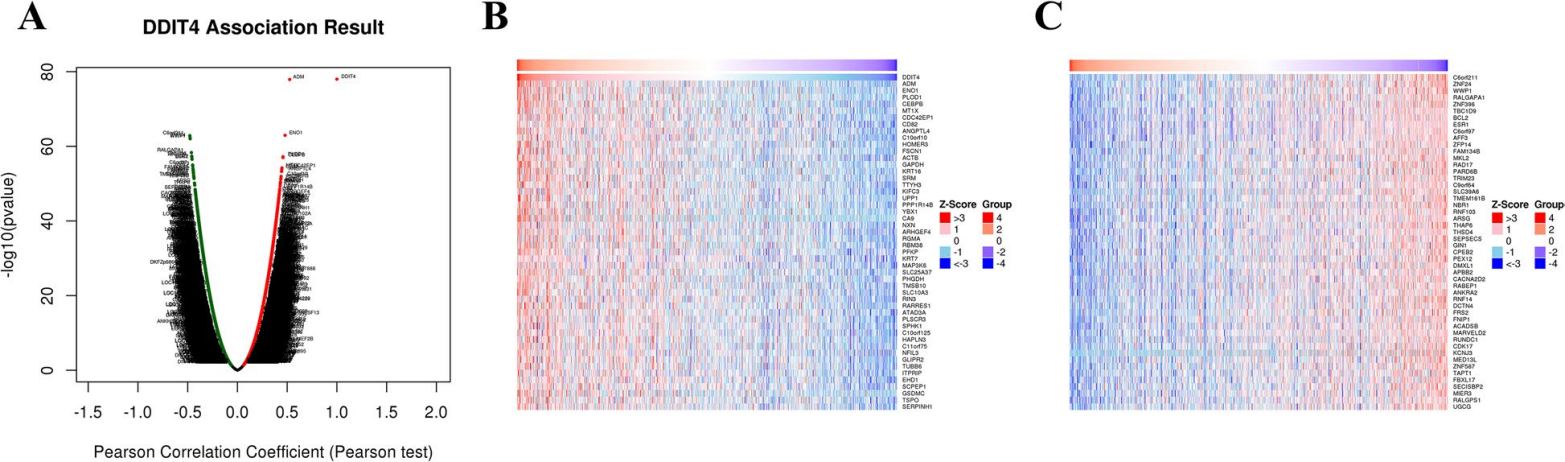
Results of the co-expression analysis of DDIT4. **A** The genes exhibiting positive and negative expression correlation with DDIT4 in breast cancer. Heatmaps showing the top 50 genes exhibiting positive (**B**) and negative (**C**) expression correlation with DDIT4 in breast cancer. Red indicates the positively correlated genes and blue indicates the negatively correlated genes

To gain additional insights into the biological significance of DDIT4, we investigated the potential role of DDIT4 in breast cancer by analyzing the mRNA sequencing data of 1093 patients with breast cancer, obtained from the TCGA database, using the LinkFinder module in LinkedOmics. As shown in Fig. 10, 7047 genes (red dots) showed positive correlation with DDIT4, whereas 5472 genes (green dots) showed negative correlation (Fig. 10A). In addition, the heatmaps showed the top 50 important genes exhibiting positive and negative co-expression with DDIT4 in breast cancer (Fig. 10B, C). Moreover, the top four significant genes, namely *ADM* (Cor = 0.276, *p* = 1.242e − 78), *ENO1* (Cor = 0.229, *p* = 1.149e − 63), *PLOD1* (Cor = 0.210, *p* = 6.321e − 58), and *CEBPB* (Cor = 0.210, *p* = 9.956e − 58) were considered as hub genes; the expression of these genes was strongly associated with DDIT4 expression in breast cancer.

### Analysis of the hub genes of DDIT4 in breast cancer

**Fig. 11:**
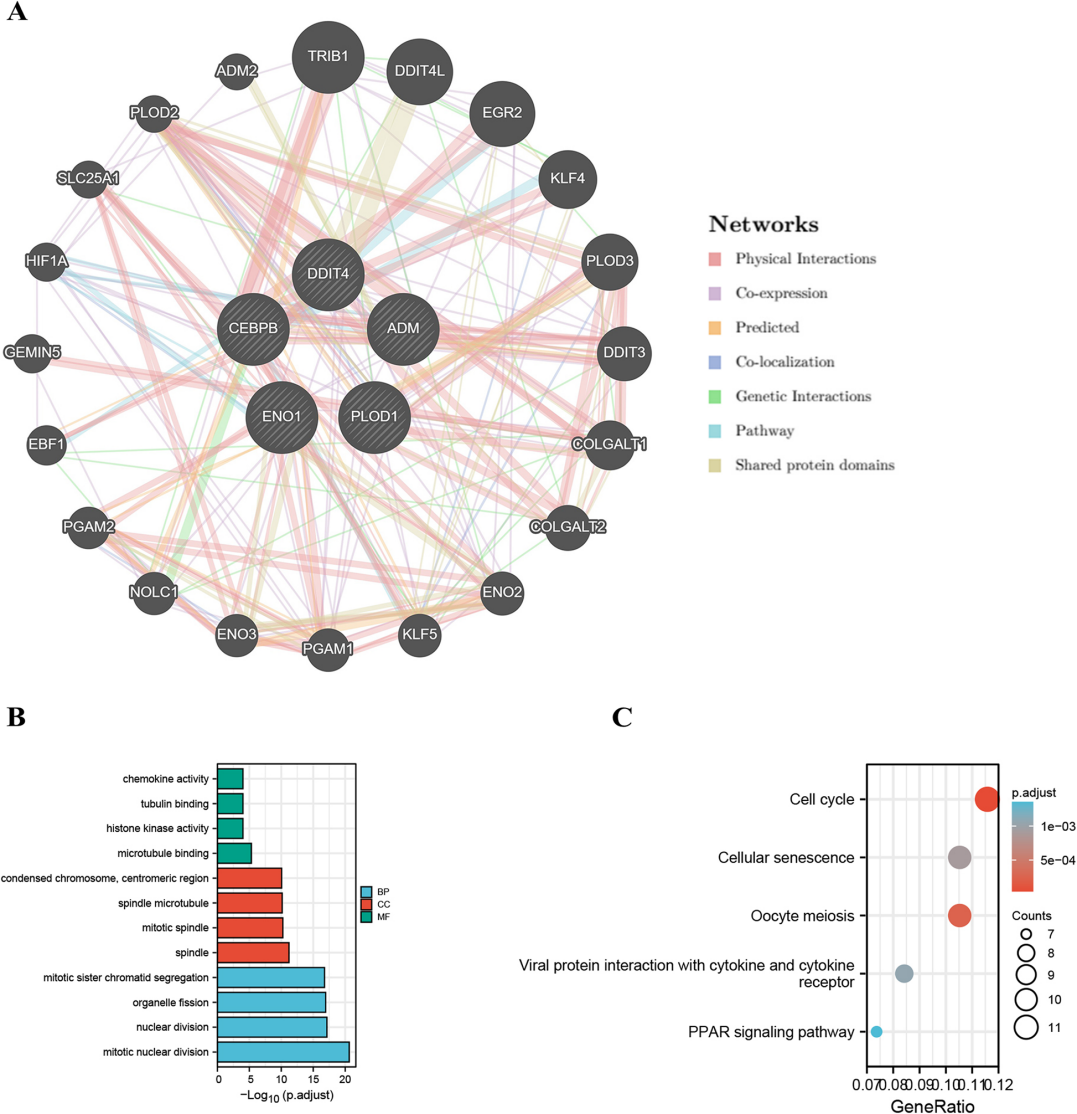
**A** PPI network of DDIT4 and hub genes produced using GeneMANIA. **B** The GO items for the genes with expression associated with DDIT4 expression. **C** The KEGG items for the genes with expression associated with DDIT4 expression

To further explore the function of DDIT4 and its hub genes in greater detail, we constructed PPI networks using the GeneMANIA tools. DDIT4 and its hub genes showed interactions with 20 genes (Fig. 11A). GO analysis revealed that the genes associated with DDIT4 are primarily related to chemokine activity, tubulin binding, and histone kinase activity and involved in physiological processes such as condensed chromosome, centromeric region, and spindle microtubule. Their molecular functions include mitotic sister chromatid segregation, organelle fission, and nuclear division (Fig. 11B). KEGG analysis showed that DDIT4 may play a crucial role in the development and progression of BC by participating in cellular senescence, oocyte meiosis, cell cycle, and PPAR signaling pathways (Fig. 11C).

**Fig. 12:**
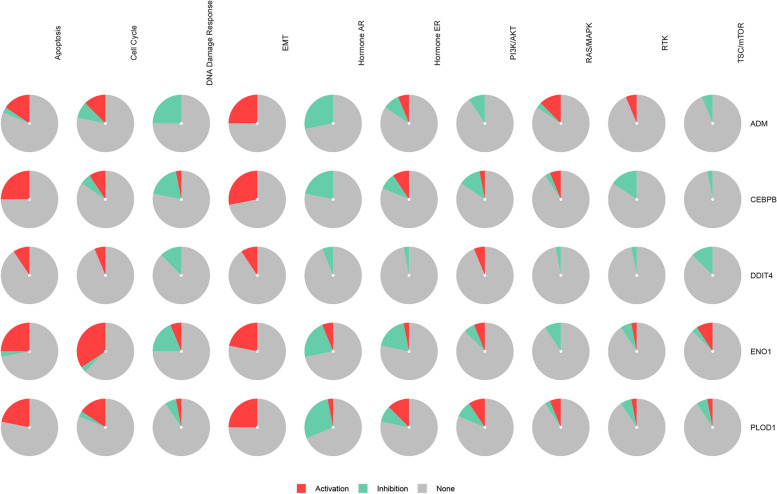
The role of DDIT4 and hub genes in the cancer-related pathways (GSCALite)

**Fig. 13:**
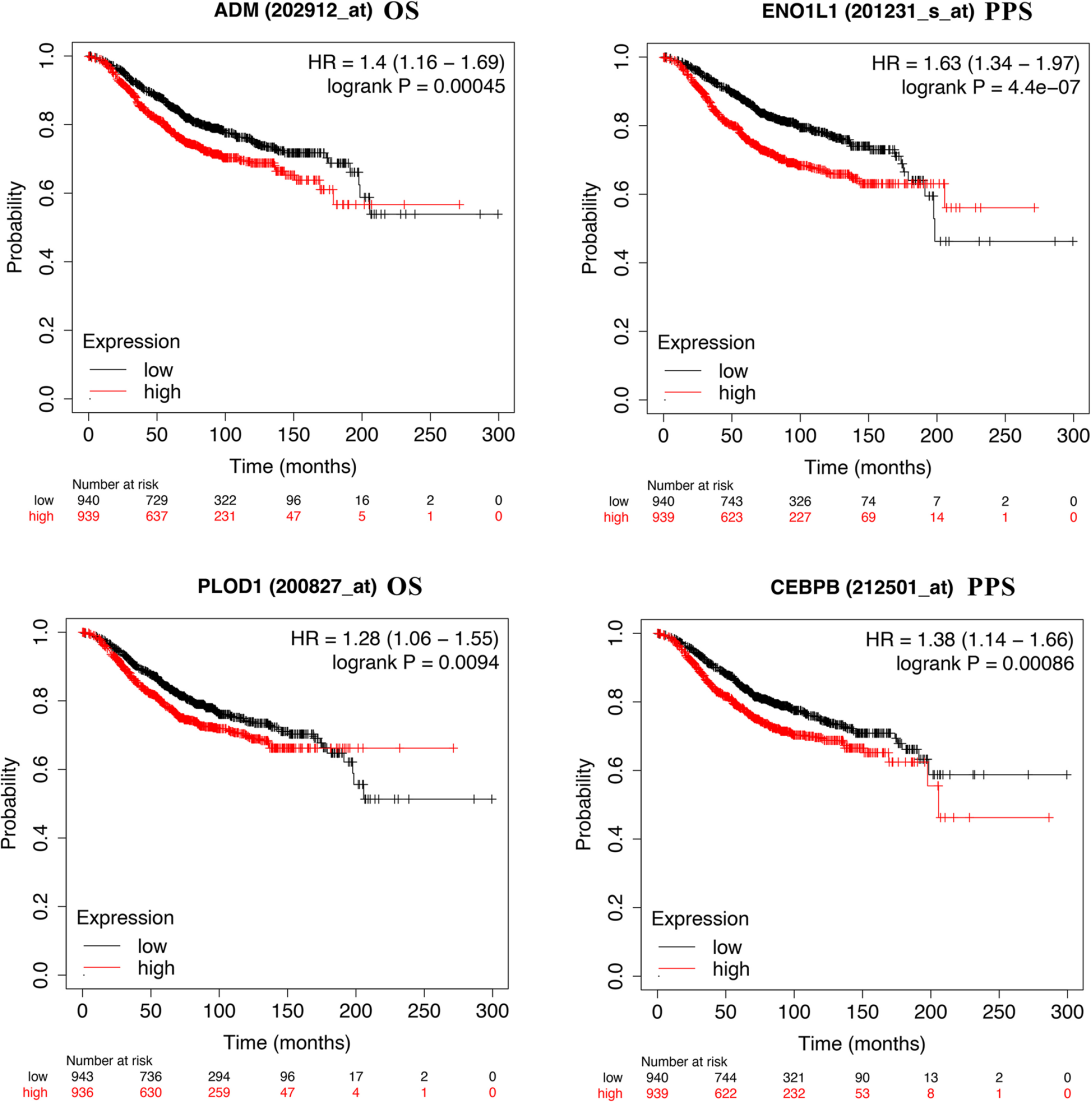
Survival analysis (OS) based on ADM, ENO1, PLOD1, and CEBPB expression in patients with breast cancer obtained from KM plotter

Furthermore, we demonstrated that DDIT4 and its hub genes participate in the activation of the apoptosis, cell cycle, and EMT pathways (Fig. 12). Finally, KM Plotter analysis of the hub genes showed that the high expression of ADM, ENO1, PLOD1, and CEBPB was significantly correlated with a shorter OS and poor prognosis in patients with breast cancer (Fig. 13).

## Discussion

Chemotherapy remains the standard treatment for TNBC. However, in recent years, immune checkpoint inhibitors (ICIs) have exhibited a sustained clinical response in various tumor types, including breast cancer [39]. Although TNBC, a highly heterogeneous and clinically aggressive form of tumor, has been shown to respond to ICIs, its clinical response rate is far from satisfactory [40]. Therefore, the combination of ICIs with other types of therapeutic regimens, including chemotherapy, is a significant concern in the management of TNBC. Recently, the Keynote 522 (NCT03036488) trial reported that the combination of pembrolizumab with neo-adjuvant chemotherapy improves the pathological complete response (pCR) rate in patients with TNBC [11]. Similarly, a phase III IMpassion130 clinical trial also showed that nab-paclitaxel plus atezolizumab increased the progression-free survival (PFS) and overall survival (OS) of patients with metastatic TNBC [41]. However, the phase III IMpassion131 clinical trial yielded conflicting results, showing that atezolizumab and paclitaxel could not be used successfully to achieve the primary end point [12]. On the contrary, the findings of the latest Keynote 355 (NCT02819518) trial showed that the combination of pembrolizumab with chemotherapy led to a significantly longer survival in patients with PD-L1-positive TNBC [10]. These seemingly contradictory results indicate that cancer cells may evolve rapidly under the selection of chemotherapy and ICIs. However, the underlying mechanism remains unknown, and the identification of novel biomarkers for patient stratification and prognosis is an urgent need.

In this study, we performed the RNA-seq of docetaxel- and doxorubicin-treated TNBC cells and sought to explore new genes that potentially contribute to the regulation of chemoresistance and immune response in TNBC. Based on the comparison of data from GO and KEGG analyses and DEG enrichment between the drug-treated and control TNBC cell groups, we found that several cellular process and networks were enriched in the docetaxel- and doxorubicin-treated TNBC cells, including cell motility, cellular metabolic process, collagen-containing extracellular matrix, mTOR signaling pathway, p53 signaling pathway, oxidative phosphorylation, and PI3K − Akt signaling pathway (Figs. 1, 2, 3, and 4). Notably, the enrichment of DDIT4 expression in TNBC cells treated with docetaxel and doxorubicin was an interesting finding for further investigation because the gene plays a key role in cancer initiation and progression as well as in stress responses, such as those to DNA damage, hypoxia, and chemotherapy [42]. Evidence from several recent studies has indicated that the overexpression of DDIT4 is also an adverse factor in ovarian carcinoma [23], gastric cancer [43], and lung adenocarcinoma [44].

In the present study, the overexpression of DDIT4 was detected in approximately half of the pan-cancer datasets in the TIMER database (Fig. 5), and the expression of this gene showed the greatest difference between normal breast and tumor tissues. We also found that the overexpression of DDIT4 was significantly correlated with different tumor stages, patient age, primary subtypes, major subclasses with TNBC, nodal metastasis status, and TP53 mutation status (Fig. 6), indicating a strong correlation between DDIT4 overexpression and breast cancer progression. Further analysis revealed that DDIT4 expression data from the TCGA effectively predicted the RFS of patients with TNBC (Fig. 7). Similarly, DDIT4 expression is associated with progression and poor survival in breast cancer, which is consistent with findings from a previous report by Pinto et al. [24]. In summary, DDIT4 may represent a promising biomarker for survival prediction in patients with TNBC.

To obtain further insights into the impact of DDIT4 overexpression on the immune microenvironment, we further analyzed the correlation of DDIT4 expression with the infiltration of immune cells in TNBC from the data published in the TIMER database. According to the analysis of data from TIMER, the abnormal expression of DDIT4 may alter the tumor microenvironment and immune response, which can significantly affect clinical outcomes. We confirmed that the overexpression of DDIT4 was associated with decreased immune cell infiltration in TNBC (Fig. 8). Although the clinical success of immune checkpoint inhibitors targeting CTLA4, PD-1, and PD- L1 has revolutionized traditional cancer treatment, response rates have remained limited, indicating that the complexity of co-evolution between cancer cells and the microenvironment may impact the response to immunotherapy. Therefore, discovery of novel biomarkers for patient stratification and application of additional immune checkpoint-related genes such as ICOS [45], LAG3 [46], TIGIT [47], BTLA [48] that control T cell function is critical for improvement of TNBC treatment. Remarkably, our findings indicated that the overexpression of DDIT4 was positively correlated with the expression of ten immune checkpoint-related genes (Fig. 9) which mainly contribute to the negative regulation of T cell activation, implying the initiation of immune evasion in tumors in response to the upregulation of DDIT4. Collectively, these results suggest that the abnormal expression of DDIT4 may contribute to the poor response to immunotherapy with ICIs, which could further induce immunotherapy resistance in TNBC.

We also identified several genes showing significant expression correlation with DDIT4, including *ADM*, *ENO1*, *PLOD1*, and *CEBPB*. The PPI network of these genes are enriched in processes and pathways related to chemokine activity, cell cycle regulation, and PPAR signaling (Fig. 11). Similarly, ENO1 can reportedly promote lung cancer metastasis via the HGFR and WNT signaling pathways [49]. PLOD1 has been shown to promote cell growth and aerobic glycolysis by regulating the SOX9/PI3K/Akt/mTOR signaling pathway in gastric cancer [50]. Through coexistence analysis, we found that DDIT4 and its hub genes are involved in the apoptosis, cell cycle, and EMT pathways.

Autophagy is an important survival mechanism that allows cells to adapt their demands to poor growth environments and maintain cellular homeostasis [51]. Evidence from numerous studies has indicated a close relationship between autophagy and anti-cancer drug resistance in breast cancer. Recently, it was reported that the inhibition of DDIT4 expression sensitizes bladder urothelial carcinoma to paclitaxel by inhibiting the DDIT4-EEF2K-autophagy axis [26]. Similarly, DDIT4 expression promoted the survival of glioblastoma cells by inhibiting mTORC1, which is a major mechanism contributing to anti-tumor therapy resistance [52]. Notably, DDIT4L, which is the paralog of DDIT4, is a p53-dependent regulator of stem cell suppression and participates in tumor migration and metastasis [53]. In addition, autophagy also facilitates tumor cell evasion from immune surveillance, leading to intrinsic resistance to antitumor immunotherapy [54]. Li et al. revealed that the high glycolytic rate in TNBC cells supports tumor-derived myeloid-derived suppressor cells (MDSCs) through the autophagy pathway [55]. Besides, major histocompatibility complex class I (MHC-I) is degraded in pancreatic cancer cells via autophagy, which promotes immune evasion [56]. Overall, the abovementioned data demonstrate that DDIT4 participates in various signaling pathways that support cancer cell survival, proliferation, immune evasion, drug resistance, and metastasis. Lastly, we found that the high expression of ADM, ENO1, PLOD1, and CEBPB is significantly correlated with the shorter overall survival of patients with BC. However, functional studied are needed to further investigate the precise mechanisms by which the products encoded by DDIT4 hub genes mediate resistance to chemotherapy and immunotherapy and to further confirm whether these hub genes are the potential indicators of worse prognosis in breast cancer.

This study had several limitations. First, the research was based solely on transcriptomic and bioinformatic analysis, and the potential biological mechanism needs further investigation and application to other types of cancers. Second, we intend to further investigate the protein expression level of DDIT4 and its role in the pathogenesis and progression of TNBC.

To summarize, we demonstrated a potential association between DDIT4 gene expression and the immunosuppressive microenvironment in TNBC. Results from this study can help identify potential biomarkers and targets for overcoming drug resistance and facilitating the clinical management of TNBC.

## Data Availability

The datasets presented in this study can be found in online repositories. The names of the repositories and accession numbers are provided in the article.
